# Effectiveness and cost of integrated cognitive and balance training for balance and falls in cerebellar ataxia: a blinded two-arm parallel group RCT

**DOI:** 10.3389/fneur.2023.1267099

**Published:** 2024-01-19

**Authors:** Stanley J. Winser, Anne Y. Y. Chan, Susan L. Whitney, Cynthia H. Chen, Marco Y. C. Pang

**Affiliations:** ^1^Department of Rehabilitation Sciences, The Hong Kong Polytechnic University, Kowloon, Hong Kong SAR, China; ^2^Division of Neurology, Prince of Wales Hospital and Department of Medicine and Therapeutics, Chinese University of Hong Kong, Hong Kong SAR, China; ^3^School of Health and Rehabilitation Sciences, University of Pittsburgh, Pittsburgh, PA, United States; ^4^Saw Swee Hock School of Public Health (Primary), National University of Singapore, Singapore, Singapore

**Keywords:** cerebellar ataxia, dual-task, dynamic balance, postural stability, cost, falls

## Abstract

**Background:**

In patients with cerebellar ataxia (CA), dual-tasking deteriorates the performance of one or both tasks.

**Objective:**

Evaluate the effects of 4 weeks of cognitive-coupled intensive balance training (CIBT) on dual-task cost, dynamic balance, disease severity, number of falls, quality of life, cognition and cost among patients with CA.

**Methods:**

This RCT compared CIBT (Group 1) to single-task training (Group 2) among 32 patients with CA. The intervention included either dual-task (CIBT) or single-task training for 4 weeks followed by 6 months of unsupervised home exercises. Dual-task timed up-and-go test (D-TUG) assessed dual-task cost of the physical and cognitive tasks. Assessment time points included baseline 1 (Week 0:T1), baseline 2 (Week 6:T2), post-intervention (Week 10:T3), and follow-up (Week 34:T4).

**Results:**

Compared to single-task training CIBT improved the dual-task cost of physical task [MD −8.36 95% CI (−14.47 to −2.36, *p* < 0.01), dual-tasking ability [−6.93 (−13.16 to −0.70); *p* = 0.03] assessed using D-TUG, balance assessed using the scale for the assessment and rating of ataxia (SARAbal) [−2.03 (−4.04 to −0.19); *p* = 0.04], visual scores of the SOT (SOT-VIS) [−18.53 (−25.81 to −11.24, *p* ≤ 0.01] and maximal excursion [13.84 (4.65 to 23.03; *p* ≤ 0.01] of the Limits of Stability (LOS) in the forward direction and reaction time in both forward [−1.11 (−1.42 to −0.78); *p* < 0.01] and right [−0.18 (0.05 to 0.31); *p* < 0.01] directions following 4 weeks of training. CIBT did not have any additional benefits in reducing the number of falls, or improving disease severity, quality of life and cognition. The mean cost of intervention and healthcare costs for 7 months was HKD 33,380 for CIBT group and HKD 38,571 for single-task training group.

**Conclusion:**

We found some evidence to support the use of CIBT for improving the dual-tasking ability, dual-task cost of physical task and dynamic balance in CA. Future large fully-powered studies are needed to confirm this claim.

**Clinical trial registration:**

https://clinicaltrials.gov/study/NCT04648501, identifier [Ref: NCT04648501].

## Introduction

Cerebellar ataxia (CA) is not a disease but instead describes a collection of symptoms associated with both genetic and acquired diseases that affect the cerebellum or its connections. CA is characterized by postural and gait instability, a lack of coordination of the extremities and trunk, and cognitive impairment (1). The overall prevalence of the diseases associated with CA is 8.22 per 100,000 population (2). Spinocerebellar ataxia has a prevalence ranging from 0.9 to 3.0 per 100,000 population (3). The overall burden of CA is high, with an annual mean cost of EUR 18,776 per patient in Spain (4) and a 6-month mean cost of HKD 146,832 in Hong Kong (HK) (5).

Poor balance and walking difficulties are common symptoms of diseases associated with CA (6), and frequent falls among individuals with CA result in significant burdens for both the individual and the healthcare system (5). The fall rate, defined as at least one accidental fall within the past 12-month period, is estimated at 93% among individuals diagnosed with CA (7). Rehabilitation exercises are considered the first-line treatment for patients with CA (8), and evidence supports the ability of rehabilitation exercises to improve balance and reduce disease severity (9). However, the limited number of high-quality studies, combined with heterogeneity among the diseases resulting in CA, have prevented the development of evidence-based guidelines for rehabilitative interventions (6). Although patients with CA show limited motor re-learning (10), some evidence supports the occurrence of motor re-learning when individuals with CA engage in repeated practice (11, 12). Understanding the potential for motor re-learning among individuals with CA is critical for designing appropriate therapeutic interventions for this population.

Dual-tasking describes the simultaneous performance of two tasks (13). The cerebellum is found to play a significant role in controlling such neural networks during the task (14). Dual-tasking deteriorates the performance of either or both tasks, which is referred to as the dual-task cost (15) among patients with neurological disorders (16, 17). In individuals with CA, dual-tasking is associated with an increased risk for falls (18) and worsen gait disturbances (19). Clinical studies of patients with CA have found that adding cognitive demands to a physical task increases the dual-task cost (19, 20). Balance training that involves dual-tasking reduces the dual-task cost among patients with stroke (21), Parkinson's disease (22), and traumatic brain injury (23).

Currently available interventions for improving balance and gait among individuals with CA do not address the potential benefits of combined intensive balance training and cognitive training in this population. Dual-task training improves dual-tasking in patients with neurological disorders other than CA (22, 23), and dual-task training in patients with CA has been suggested based on these prior findings (18, 19). Studies in the past have examined the influence of dual-tasking on balance and gait variables in this population (18, 24, 25). Studies evaluating the effectiveness of dual-task training in patients with CA are limited (19). The work by Selim et al. on the effectiveness of dual-task training on stability and function is restricted to children aged 5 to 10 years (26). Therefore there is a need for conducting an experiment that tests the benefits of dual-task training among adult patients with CA. We designed a cognitive-coupled intensive balance training (CIBT), an intervention that couples intensive balance with cognitive training, and previously found that CIBT is feasible and safe for patients with CA (27). In the current study, we conducted a randomized controlled trial (RCT) to evaluate the effectiveness and cost of CIBT training in a population with CA when compared with conventional single-task training, consisting of intensive balance training, coordination training, and cognitive exercise training, delivered separately. We assessed dual tasking, dynamic balance, disease severity, quality of life, cognition, and cost.

## Materials and methods

An assessor- and statistician-blinded, two-arm, parallel-group RCT comparing dual-task training (CIBT) with single-task training (conventional balance, coordination, and cognitive training delivered separately) was conducted between January 2020 and June 2021 among 32 patients with CA. Eligible participants were randomized into one of two groups: Group 1: dual-task training (CIBT, experimental group); Group 2: single-task training (conventional balance, coordination, and cognition training delivered separately; active control group). Study groups and allocation were concealed. Potential patients with CA were recruited from the Hong Kong Spinocerebellar Ataxia Association (HKSCAA). Ethics approval was obtained from the Human Subjects Ethics Sub-committee of HK (Ref: HSEARS20190322001). The trial was registered with clinicaltrials.gov before the onset of data collection (Ref: NCT04648501).

Study participants were randomized into one of the two intervention groups before the first baseline assessment using permuted blocks determined using computer-generated random numbers. Allocation was performed by a researcher who was blinded to recruitment, assessment of study variables, or intervention delivery. One HK-registered physiotherapist delivered the intervention for both groups. All assessments were performed by individuals blinded to group allocation. We conducted two baseline assessments (T1 and T2) to allow for the prospective evaluation of the number of falls and disease severity progression during a 6-week period. The first baseline assessment (T1) was completed after obtaining written informed consent and demographic data. Participants were then requested to return after 6 weeks for the second baseline assessment (T2), at which point all assessment variables were evaluated, including fall history over the past 6 weeks. The post-intervention assessment was performed after 4 weeks of supervised intervention exercises (T3), and a follow-up assessment was performed after 6 months of unsupervised home practice of intervention exercises (T4).

The following inclusion criteria were applied: (1) individuals of both genders aged 18–60 years; (2) individuals with a confirmed diagnosis of CA (of any type); and (3) individuals able to walk independently with or without assistive walking aids. The following exclusion criteria were applied: (1) any previous history of other neurological diseases (such as Parkinson's disease, stroke, or polyneuropathies) or musculoskeletal problems severely impairing balance, gait, or motor performance; (2) wheel-chair or bed-bound patients who can walk only with handheld support; (3) severe visual impairment preventing exercise participation; or (4) severe cognitive impairment, defined as a score <16 on the Montreal Cognitive Assessment (MoCA) scale (28).

Treatment was initiated for both groups after T2 and continued for 7 months, including 4 weeks of supervised training at HK Polytechnic University (PolyU) and 6 months of unsupervised home training. For the first 4 weeks, both groups attended 60-min training sessions at PolyU, 3 times a week for 4 weeks. After the initial 4-week training phase, participants were asked to complete unsupervised home exercise programmes consistent with their intervention group assignments for the next 6 months.

Training sessions for the experimental, dual-task CIBT group (Group 1) consisted of 10 min of warm-up, 40 min of CIBT training, and 10 min of cool-down. The CIBT programme involves the performance of four types of cognitive tasks during the following physical tasks: sit-to-stand; standing with feet apart; one leg, tandem standing; multidirectional reaching; stair climbing; and walking 10 m. The details of the cognitive tasks are reported elsewhere (27). Motor–cognitive interactions occur when highly challenging cognitive tasks are performed simultaneously with physical tasks, increasing the risk of falls among individuals with CA (19). A careful calibration of the cognitive task difficulty level is necessary to ensure the safety of participants. Each participant's tolerance for motor–cognitive interactions was assessed individually, and the initial difficulty level and progression of both cognitive and physical tasks were determined for each individual to ensure safety.

Treatment sessions for the single-task training group (active control, with conventional balance training, coordination training, and cognitive training were delivered separately; Group 2) consisted of 10 min of warm-up, 20 min of conventional balance and coordination exercises in line with previously published literature (29), 20 min of single-task cognitive training (using the same four tasks used during CIBT), and 10 min of cool-down. In addition, fall prevention strategies were also taught for both the groups. In summary, the CIBT group had 40 min of dual-task training and the control group had the same 40 min of similar exercises included in the CIBT but were delivered as single-task training. For the 6-months follow-up period, the home-based exercises were similar to the exercises delivered during the 4-week intervention phase. The participants were handed pamphlets summarizing the exercises. The dosage of exercises during the follow-up period for both groups was similar however, they were unsupervised.

The progression of treatment for both groups was determined by the Physiotherapist delivering the intervention. The type of physical task and the cognitive task were tailor-made that suit the capacity of the participants. Treatment progression was done once every week. The physical task progression principles included reducing the base of support, physical support, verbal cues, altering the support surface, and changes in the speed of the activity such as walking slower or faster. The progression for cognitive tasks included changes in the difficulty of arithmetic calculation, difficulty of memory tasks and complexity of cognitive tasks such as recollecting rare vegetables or seasonal fruits.

### Primary outcome measure

The dual-task costs of physical and cognitive tasks were assessed using the timed up-and-go test (TUG). The standard, single-task TUG (30); the dual-task TUG (D-TUG); and counting backwards were assessed consecutively. During the D-TUG, participants were instructed to count backwards by four from a random starting number while performing the standard TUG. The time to complete the task (in seconds) was recorded. The time required (in seconds) to count backwards by fours from the same starting number without performing a physical task was also recorded. The dual-task cost of physical tasks was estimated using the following formula: [(D-TUG – Standard TUG) ÷ Standard TUG] × 100 (31). The dual-task cost of cognitive tasks was assessed using the following formula: [(D-TUG – Standard backward counting backwards) ÷ Standard counting backwards] × 100. The scores of the DTUG was used to assess dual-tasking ability.

### Secondary outcome measures

We included standardized and validated measures for assessing functional balance [Berg Balance Scale (BBS) (30) and Scale for the Assessment and Rating of Ataxia (SARA) balance component (SARAbal) (30)], dynamic stability (LOS) (32, 33), sensory interaction (SOT) (33, 34), number of falls, cognitive function (MoCA) (35), ataxia severity (SARA) (30, 36), and number of falls and quality of life (EuroQol-5 dimension-5 level [EQ-5D-5L]) (37). A summary of the proposed outcome measures, the domains tested, interpretations, and the assessment timeline are reported elsewhere (27). To ensure the rigor of the balance assessment we have used the International Classification of Function (ICF) model in choosing the assessents (38). Body structure and function level assessment of balance identifying the underlying impairment is reported using the DTUG, SOT, and LOS. Activity level assessment of balance is reported using the BBS and TUG. The SARA and SARA-bal are disease-specific measures appropriate for patients with CA (32).

### Adherence

Adherence to the intervention protocol was monitored using electronic diaries to reduce missing values and recall errors. At the end of the 4-week supervised training session, participants were provided information on how to access and complete the electronic diaries.

### Cost estimation

Cost was assessed using digital or manual self-reported questionnaires. Each participant's digital diary interface was encoded with a unique identification number to ensure privacy. Participants were instructed to complete the digital diary once per month. The researcher interface of the digital diary provided a summary of completed items for all participants. The research assistant followed up with non-responders every month by phone. A printed version of the cost and fall (manual) diary was provided to participants with limited access to the internet. Postage-paid envelopes were included with each printed cost diary to obtain a better response rate, and participants were instructed to post the completed forms once per month. Table 1 lists the direct medical costs. The derived costs of items relevant to the healthcare perspective are reported as the unsubsidised rate for medical care for 2018 (in HKD).

**Table 1:** Cost sheet.

**Direct cost**	**Unsubsidized price per unit**
Cost of intervention	HKD 250
Consultation with GP	HKD 445
Visit to specialist (Geriatric day hospital)	HKD 1,960
Specialist outpatient	HKD 1,190
Consultation with TCM	HKD 445
Accident and emergency department contact following falls	HKD 1,230
Hospitalization charge per day (admission plus fees)	HKD 5,100/day
Intensive care ward/unit	HKD 24,400/day
Surgical intervention following falls	Variable
Drugs	Variable
Dressing or injection	HKD 100
Professional home care	HKD 535
Community allied health service	HKD 1,730
Aids and appliances	Variable

Source for cost sheet: 1. Hospital Authority. Fees and charges. Available online at: http://www.ha.org.hk/visitor/ha_visitor_index.asp?Parent_ID=10044&Content_ID=10045&Ver=HTML (accessed February 9, 2018). 2. Localiiz. Public or Private? A Guide to Healthcare in Hong Kong. Localiiz the Site with Insight (2017). Available online at: http://hk.localiiz.com/public-or-private-a-comprehensive-guide-to-healthcare-in-hong-kong/#.WmkrfYVOJR (accessed January 25, 2018).

### Statistical analyses

The analysis was performed according to the intention-to-treat principle. Maximum likelihood imputation was used to generate data for missing items. Demographic data are reported as the mean and standard deviation (SD) for continuous variables and as the number and percentage for categorical variables. To establish baseline differences between groups, we applied the independent *t*-test for continuous variables and the Chi-square test for categorical variables. Variables without a normal distribution were log-transformed prior to analyses. The mean attendance percentage for the treatment sessions was calculated as the percentage of training sessions attended by the participants against the total number of planned treatment sessions for both supervised (institution-based) and unsupervised (home-based) training sessions. Changes in primary and secondary outcome measures (evaluated at T2, T3, and T4) across the intervention groups were evaluated using an analysis of covariance (ANCOVA) at a 95% confidence interval (CI). Baseline scores were adjusted for disease duration, ataxia severity, and assistive walking device use (co-variates). T2 and T3 were compared to establish immediate treatment effects, whereas T3 and T4 were compared to evaluate the long-term effects of the intervention. Considering the large number of comparisons, to minimize the likelihood of type 1 error Bonferroni correction was included in the analysis. Effect sizes (Cohen's F) were computed using the formula [$\surd \overset{´}{\eta }$p^2/^(1 – $\overset{´}{\eta }$p^2^)], where $\overset{´}{\eta }$p^2^ is the partial eta-squared value obtained from the analysis of covariance. The effect size was interpreted as small if the value of Cohen's F was 0.10 or below, moderate at 0.25, and large at 0.40 or above (39). The mediation effects of dual-task cost of the physical and cognitive task on reducing the number of falls were assessed using the Sobel test. The mean cost of the intervention and health care costs through the follow-up period are reported as the mean and SD. The EQ-5D-5L response was converted into a utility score used to estimate the gain or loss in quality-adjusted life-years during the follow-up period. The cost-effectiveness of the intervention will be reported in a subsequent paper.

## Results

**Figure 1:**
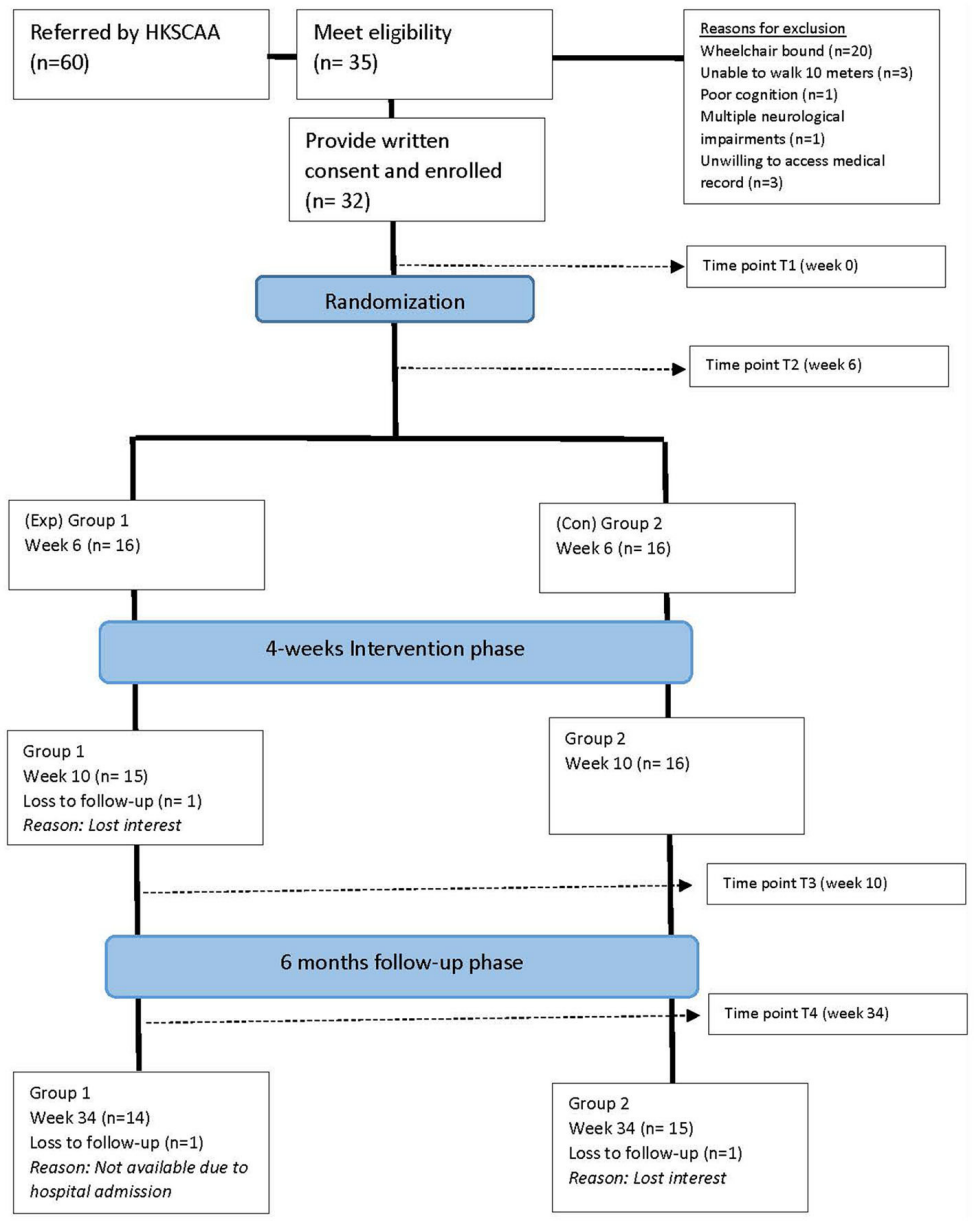
Flow of the study. HKSCAA, Hong Kong Spinocerebellar Ataxia Association.

Sixty potential participants were approached through the HKSCAA between January 2019 and December 2021. Of these, 32 participants were deemed eligible and consented to participate. Enrolled participants were randomized into the experimental (Group 1, *n* = 16) and control groups (Group 2, *n* = 16) after the first baseline assessment (T1). At the end of the 4-week intervention phase (T3), one participant from Group 1 was lost to follow-up. One additional participant from the Group 1 and one participant from the Group 2 withdrew from the study during the 6-month follow-up assessment (T4). The withdrawal was not related to the study intervention. Figure 1 illustrates the flow of this study.

The baseline characteristics of study participants are presented in Table 2. The mean age of study participants was 48 years (SD: 10.2 years), and 60% of enrolled participants had a subtype of spinocerebellar ataxia, whereas the remaining participants had either degenerative or idiopathic ataxia. The demographic characteristics were insignificant between the groups at baseline. We found insignificant differences in ataxia severity scores (SARA) and ataxia-specific balance scores (SARAbal) between the two baseline assessments (T1 and T2), indicating that participants remained stable in terms of disease severity. The mean attendance percentage for institution-based intervention sessions across both groups was 85%, but it dropped to 62% during the 6-month follow-up period. No adverse events, such as falls, were encountered during the intervention phase. Less than 25% (7 of 32) of participants reported mild muscle pain following the first two intervention sessions, and the pain subsided completely within a few days. The intervention was well-tolerated by all participants across both groups. The mean scores of all outcome measures at all four assessment time points can be found in Appendix 1.

**Table 2:** Demographics of the participants (*n* = 32).

**Total no. of participants**			**Group 1 (*n =* 16) (CIBT)**	**Group 2 (*n =* 16) (Control)**	***p***
Age Mean (SD)	Mean (SD)		50.20 (14.41)	46.00 (14.05)	0.75
	Range		32–75	26–75	
Gender	Male		8 (50%)	10 (56%)	0.87
	Female		8 (50%)	8 (54%)	
Ethnicity	Chinese		16 (100%)	16 (100%)	1
Occupation	Employed		8 (50%)	9 (56%)	0.52
	Unemployed		4 (25%)	3 (19%)	
	Retired		4 (25%)	4 (25%)	
Diagnosis	Spinocerebellar ataxia	SCA-1	2 (12%)	0 (0%)	0.91
		SCA-3	8 (50%)	7 (44%)	
		SCA-11	0 (0%)	2 (12%)	
	Post-infectious cerebellar degeneration		1 (6%)	1 (6%)	
	Unknown cause for ataxia		5 (31%)	6 (38%)	
Age at disease onset	Mean (SD)		37.70 (14.31)	30.67 (8.29)	0.24
	Range		19–56	17–40	
Disease duration	Mean (SD) in years		12.50 (10.73)	15.33 (11.11)	0.35
	Range		3–36	2–38	
Use of assistive walking device	Yes		14 (88%)	13 (81%)	0.92
	No		2 (12%)	3 (19%)	

SCA, Spinocerebellar ataxia.

### Effect of exercise on dual tasking

Compared to single-task training the CIBT resulted in a significant improvement in dual-tasking ability [MD −6.18, 95% CI (−10.93 to −1.45; *p* = 0.01] and dual-task cost of physical task [MD −8.36 95% CI (−14.47 to −2.36, *p* < 0.01) assessed using the DTUG immediately following the intervention. The effect size of these changes ranged from small to moderate. A summary of between-group differences with 95% confidence interval of all outcome measures is reported in Table 3. A summary of within-group differences at post-intervention and follow-up assessment for all the outcome measures is reported in Appendix 2.

**Table 3:** Between-group mean difference and 95% confidence interval across assessment time points T3 (post-intervention) and T4 (follow-up).

**Outcome measure**		**Time points**	**MD (95% CI)**	***P* value**	**Partial eta squared**	**Effect size (Cohen's F)**
**Measures of dual–tasking and balance**						
Dual-task-cost (Physical task)		T3	−8.36 (−14.47 to −2.26)	<0.01^*^	0.09	0.30
		T4	−6.99 (−13.26 to −0.73);	0.03^*^		
Dual-task-cost (Cognitive task)		T3	−14.80 (−33.94 to 4.33)	0.12	0.005	0.07
		T4	−11.95 (−27.41 to 3.52)	0.12		
Dual-task TUG		T3	−6.93 (−13.16 to −0.70)	0.03^*^	0.09	0.30
		T4	−7.99 (−0.08 to 16.07)	0.06		
SARA-Bal		T3	−2.03 (−4.04 to −0.19)	0.04^*^	0.12	0.36
		T4	−0.74 (−3.99 to 2.51)	0.64		
BBS		T3	1.75 (−4.48 to 7.96)	0.57	0.01	0.10
		T4	4.16 (−3.80 to 11.94)	0.28		
SOT	SOT-SOM	T3	−4.42 (−10.90 to 2.06)	0.17	0.09	0.30
		T4	1.49 (−1.58 to 4.54)	0.32		
	SOT-VIS	T3	−18.53 (−25.81 to −11.24)	<0.01^*^	0.64	1.3
		T4	−16.94 (−23.44 to −10.44)	<0.01^*^		
	SOT-VEST	T3	−0.89 (−6.53 to 4.75)	0.74	0.05	0.22
		T4	−2.54 (−11.01 to 5.91)	0.54		
	SOT-COMP	T3	9.03 (−1.32 to 19.39)	0.8	0.09	0.30
		T4	0.06 (−9.61 to 9.73)	0.99		
LOS	LOS-F-RT	T3	−1.11 (−1.42 TO −0.78)	<0.01^*^	0.50	1
		T4	−0.69 (−0.94 TO −0.44)	<0.01^*^		
	LOS-F-MXE	T3	13.84 (4.65 to 23.03)	<0.01^*^	0.32	0.68
		T4	21.01 (9.07 to 32.95)	<0.01^*^		
	LOS-R-RT	T3	−0.18 (0.05 to 0.31)	<0.01^*^	0.01	0.10
		T4	−0.05 (−0.41 to 0.29)	0.73		
	LOS-R-MXE	T3	−0.17 (−25.56 to 22.12)	0.88	0.01	0.10
		T4	−2.70 (−11.65 to 6.24)	0.54		
	LOS-B-RT	T3	−0.51 (0.22 to 0.78)	<0.01	0.05	0.22
		T4	−0.03 (−0.35 to 0.42)	0.19		
	LOS-B-MXE	T3	−8.35 (−28.42 to 11.72)	0.40	0.01	0.10
		T4	4.41 (−6.13 to 14.92)	0.39		
	LOS-L-RT	T3	0.14 (−0.79 to 0.35)	0.20	0.01	0.10
		T4	0.03 (−0.30 to 0.36)	0.84		
	LOS-L-MXE	T3	2.27 (−17.41 to 21.95)	0.81	0.01	0.10
		T4	9.41 (0.34 to 18.49)	0.04^*^		
**Measures of disease severity, quality of life and cognition, mean (SD)**						
SARA		T3	−3.41 (−7.01 to 0.18)	0.17	0.08	0.29
		T4	−2.87 (−7.09 to 1.34)	0.47		
HK-MOCA		T3	−0.61 (−3.65 to 2.43)	0.68	0.01	0.10
		T4	0.80 (−2.09 to 3.70)	0.57		
EQ-5D-5-L	EQ-VAS	T3	−5.43 (−13.40 to 2.53)	0.17	0.10	0.33
		T4	−5.32 (−10.88 to 0.25)	0.06		
	QALY	T3	0.04 (−0.07 to 0.16)	0.49	0.01	0.10
		T4	−0.02 (−0.13 to 0.09)	0.71		

The mean difference is expressed as group 1 (CIBT)- group 2 (Control). T3, assessment timeline 3; T4, assessment timeline 4; RT, reaction time; MXE, Maximal excursion; SOT, Senosry Organization Tesst; Som, somatosensory; Vis, visual; Vest, vestibular; Comp, composite; BBS, Berg balance scale; SARA-bal, Balance sub-component of the scale for the assessment and rating of ataxia; R, right; F, front; L, left; B, back; RT, Reaction time; MXE, Maximal excursion; LOS, Limits of stability; EQ-5D-5L, Euro-qol 5 dimension, 5 level; EQ-VAS, visual analog scale of the Euro Qol questionnaire; QOL, Quality-adjusted life year; SARA, scale for the assessment and rating of ataxia; TUG, Timed up and go test; HK-MOCA, Montreal Cognitive Assessment Scaale; Hong Kong version.

### Effect of exercise on dynamic balance

Compared to single-task training the CIBT significantly improved dynamic balance assessed using SARAbal [−2.03 (−4.04 to −0.19); *p* = 0.04], dynamic stability assessed using maximal excursion [13.84 (4.65 to 23.03); *p* ≤ 0.01] and reaction time [−1.11; (−1.42 to −0.78); *p* < 0.01] of the LOS test in the forward direction and reaction time [−0.18 (0.05 to 0.31); *p* < 0.01] in the right direction of the LOS. We found a significant reduction in visual scores of the SOT (SOT-VIS) [−18.53 (−25.81 to −11.24), *p* ≤ 0.01] among the CIBT group suggestive of a reduction in visual reliance following 4 weeks of CIBT. The effect size of these changes ranged from moderate to large. Benefits attained were not retained for any of these balance measures during the follow-up assessment.

### Effect of intervention on falls

We did not find significant differences between the two groups for the percentage of fallers, the number of falls per person per month, or the number of near falls per person per month at both post-intervention (T3; Table 4) and at the end of the 6-month follow-up phase. The relative effects of the intervention on falls, measured as the ratio between incidence rates between groups, was 0.83 (95% CI: 021–1.96; *p* = 0.21) for the number of falls in the past month and 0.95 (95% CI: 0.12–4.01; *p* = 0.32) for near falls, indicating no significant differences between groups. Considering the insignificance of the findings the mediation effect was not further explored.

**Table 4:** Comparison of 1 month falls reporting at the end of intervention phase (T3).

**Category**	**Falls rate (falls/person-1 month)**		**IRR experimental-control (95% CI)**	***p***
	**Group 1**	**Group 2**		
Number of falls	0.31	0.37	0.83 (0.21–1.96)	0.21
Number of near falls	2.68	2.81	0.95 (0.12–4.01)	0.32

IRR, Incidence ratio rate; p, level of significance.

### Effect of exercise on disease severity

No between or within-group differences were identified in disease severity assessed using SARA among the CIBT group suggestive of no additional effect of the intervention on disease severity.

### Effect of exercise on health-related quality of life and cognitive function

ANCOVA demonstrated no beneficial effect of the intervention on the health-related quality of life assessed using the EQ-5D-5L and cognitive function assessed using the MoCA at the end of 4 weeks of intervention as well as during the follow-up assessment.

### Cost

The mean cost of the intervention and the healthcare cost across 7 months is reported in Table 5. The mean cost for delivering the intervention, including the cost of hiring a registered physiotherapist, the cost of the intervention site, and the administrative costs, associated with an average of 12 sessions over 4 weeks for each group, were HKD 4,200. The total mean cost of the intervention plus the healthcare costs for 7 months was HKD 33,380 for Group 1 and HKD 38,571 for Group 2.

**Table 5:** Mean cost of the intervention and the healthcare cost of 7 months across the intervention groups.

**Cost items**	**Group 1**		**Group 2**	
	**Mean units**	**Mean cost**	**Mean units**	**Mean cost**
Cost of intervention	12	$4,200	12	$4,200
Visit to GP	4.75	$2,114	4.06	$1,808
Visit to specialist	2.13	$4,165	3.75	$7,350
Chinese medicine	2.63	$1,168	6.38	$2,837
Acupuncture	4.69	$2,086	6.5	$2,893
Hospitalization	0.94	$4,781	1.56	$5,969
Surgical expense	0	0	0	0
Medical supplies	NA	$2,633	NA	$3,220
Medical investigation	1.63	$9,962	0.25	$1,232
AHS	0	0	0	0
Rehab service	NA	$914	NA	$3,586
Transport	NA	$1,096	NA	$4,203
Home modification	NA	$261	NA	$1,273
Total cost	$33,380		$38,571	

GP, General practioner; AHS, Allied Health services; $, Hong Kong Dollars.

## Discussion

This study compared CIBT, consisting of intensive dual-task training, with conventional single-task training among patients with CA. Both groups were delivered 4 weeks of supervised exercises, followed by 6 months of unsupervised home exercises. Assessed outcomes included dual-task cost, dual-tasing ability, dynamic balance, fall rate, disease severity, health-related quality of life, and cognition. Some immediate beneficial effects were observed following the intervention on the dual-task cost of physical tasks and dual-tasking ability assessed using the DTUG. This improvement can be accounted to the contribution of the cerebellum in shifting an additionally demanding task such as dual-tasking to an automatic task with repeated practice (40, 41). The attention theory helps understand this concept where repeated practice results in an improved capacity to shift attention to the secondary task during dual-tasking (42, 43). We found some immediate effects of the intervention on the dynamic balance assessed using the SARAbal. The scale assesses the ability to walk, stand and sit unsupported. A reduction in the postural sway during sitting and standing increased the SARAbal score post-intervention. These changes are in line with previous literature (29). The positive changes captured using the SOT are suggestive of an improvement in the sensory interaction of balance following the intervention. The long-term benefits of CIBT were limited in that we found insignificant differences between groups after 6 months of home-based unsupervised exercises. CIBT did not have any additional benefits in reducing the number of falls or improving disease severity, quality of life and cognition compared to conventional single-task training. The mean cost of intervention and healthcare costs for 7 months was HKD 33,380 for group 1 and HKD 38,571 for group 2.

The dual-task cost of both physical and cognitive tasks during dual-tasking is high among individuals with CA (18, 20, 44). Evidence indicates that repeated practice of dual-tasking results in reductions in dual-task costs, resulting in improved dual-tasking ability (15). Our study also provided some evidence for the reduction in dual-task cost of physical tasks and an improvement in dual-tasking ability following CIBT. A reduction in reaction time, task automatization and optimization of attention allocation during dual-task (45) explains the reduction in dual-task cost (15). We hypothesized dual-task training (CIBT) will have an advantage over single-task training in reducing the number of falls. However, the findings of this study did not support our hypothesis in that, there were no significant between-group differences in the number of falls. We also did not find beneficial effects of CIBT compared with conventional training on quality of life. The inability to identify any significant effects of CIBT may be due to the short intervention period. An extended intervention period, such as 8 or 12 weeks, may be necessary to observe significant changes in the number of falls, quality of life and cognition. Secondly, our falls assessment captured the number of falls in the past 1 month. An ongoing falls assessment throughout the trial period may have better captured the number of falls. Lastly, the follow-up period of 6 months for the fall assessment may have been too short to capture the difference in the number of falls between the groups. Furture studies need to consider ongoing assessments for capturing fall history and longer follow-up periods to see the benefits of dual-task training on falls in this population.

Physical exercises, including sit-to-stand; standing with feet apart; one leg, tandem standing; multidirectional reaching; stair climbing; and walking, are beneficial for improving dynamic balance among individuals with CA (9, 29). The physical training tasks included for both groups included these types of exercises; therefore, we anticipated balance improvements would occur in both groups. Our findings revealed balance improvement assessed using the SARAbal and not in BBS following the intervention. SARAbal is a disease-specific measure of balance and is arguably more sensitive to changes in balance in this population when compared to the BBS. The other interesting finding in terms of dynamic balance was revealed in the laboratory-based assessment tools (SOT and LOS). We found significant between-group differences among the visual scores of SOT (SOT-VIS) and maximal excursion in the forward (MXE-F-LOS) direction and reaction time in the forward (RT-F-LOS) and right (RT-R-LOS) directions of the LOS test. These differences could be argued due to the nature of the assessment. Both these assessments require participants to follow the visual cues on the screen which mimic dual-tasking. Group A demonstrated superior dual-tasking ability during the assessment which may have resulted in better performance. In addition, the sensitivity of the LOS to identify subtle changes in dual-tasking may have been superior when compared to the BBS. Lastly, previous studies report the impairment in vestibular-ocular reflex (46), visual influence and dependence (47, 48) during dynamic tasks in this population. The findings of our study demonstrate a decrease in visual scores of the SOT post-intervention suggestive of a reduction in visual dependence during the dynamic balance task. Future studies are warranted to further examine the relationship between dual-tasking and its effects on visual dependence and vestibulo-ocular reflex in this population. The gains achieved in dynamic balance were not sustained during the follow-up assessment. Within-group differences of these outcome measures between time points T3 and T4 revealed an insignificant difference suggestive of a lack of retention of training effects following 6 months of unsupervised home exercise. The mode of exercise may have influenced the results. The participants were asked to perform unsupervised exercises for 6 months. Though measures were taken to ensure an adequate number of treatment sessions per week, the lack of direct supervision limited researchers from ensuring the adequacy of the exercise dosage. Future studies need to consider measures to ensure adequacy of treatment dosage during the follow-up period.

The CIBT exercise did not have additional benefits in improving the disease severity compared to the control. SARA is a composite score of both motor and non-motor symptoms including impairments in balance, speech, incoordination of limb, and eyeball movement. Our balance training exercises had a positive effect on gait, sitting and standing balance and dysmetria (finger-chase, dysdiadokokinesia and heel-shin slide test) of the SARA among both the groups. A mean reduction of 4 points in the SARA scale among group 1 met the minimal detectable change for intraindividual score differences of <3.5 (49). Yet additional benefits of CIBT over the control intervention on disease severity and the influence of the intervention on non-motor symptoms are still uncertain. For the cognitive assessment, our participants' average score of the MOCA is indicative of mild cognitive impairment (50). The MOCA assesses short-term memory, visuospatial abilities, executive function, attention, language, and orientation. Insignificant within and between-group differences in the scores are suggestive that dual-task training alone is not sufficient to improve for aforementioned cognitive function among patients with CA.

In contrast to our hypothesis, the cost findings across the groups were comparable. We hypothesized a greater reduction of falls rate among group 1 that will yield higher healthcare cost savings. Our follow-up of 6 months may not have been sufficient to capture the healthcare cost difference between the two groups. Our previous study reported the mean 6 monthly costs of CA among the HK population as HKD 146,832 (5). In contrast, the current study reports an average mean healthcare cost of HKD 35,975 per 7 months. The difference in the estimation is due to the difference in the cost estimation perspective. The former study was done from a societal perspective that included non-medical expenses such as loss of productivity while the present study focuses only on the healthcare perspective. Secondly, the participants included in this study are ambulant and arguably have lesser healthcare expenses when compared to the former cohort.

### Strengths and limitations of the study

Our study has several strengths. (1) We implemented multiple strategies (digital diary, follow-up phone call reminders, and routine encouragement to continue exercise) to minimize missing data, (2) rigorous methodological procedures were adopted to ensure the quality of this study, (3) the use of a standardized set of outcome measures with sound psychometric properties were used for assessing the study variables, (4) the use of ICF classification for choosing the outcome measures increased the rigor of balance assessment, and (5) additional measures were taken to ensure adequate home-based practice, including tracking the participants' attendance through the digital diary and phone calls to remind participants with poor attendance.

The findings of this study have to be interpreted with caution due to the following limitations (1) we targeted recruiting 44 participants however 12 months of data collection yielded only 32 participants making the study findings underpowered. Future studies need to consider including more public hospitals in HK and Mainland China to increase the sample size, (2) our sample did not include any acquired causes of CA and therefore generalizing the finding to all types of CA is limited, (3) our sample is restricted to ambulant patients with CA and therefore the benefits of dual-tasks training among patients with poor balance and the non-ambulant is unknown, (4) we used the past 1-month fall history to record the number of falls and we likely missed falls during the period beyond the assessment window, (5) our measures for tracking the adherence to home-exercise including digital diary and phone call reminder limited the researchers from ensuring adequate treatment dosage during the follow-up period. More objective tracking measures such as fitit, applewatch or other similar trackers need to be considered in the future, and (6) finally, our cost estimation was self-reported, though the self-reporting was completed every month the change for recall bias cannot be eliminated.

## Conclusion

This study demonstrates some evidence to support the benefits of 4 weeks of intense dual-task training on the dual-task cost of physical task, dual-tasking ability and dynamic balance. The long-term benefits of CIBT are found to be limited. CIBT did not improve falls, quality of life and cognition in this population.

## Data availability statement

The original contributions presented in the study are included in the article/Supplementary material, further inquiries can be directed to the corresponding author.

## Ethics statement

Ethics approval was obtained from the Human Subjects Ethics Sub-committee of HK (Ref: HSEARS20190322001). The studies were conducted in accordance with the local legislation and institutional requirements. The participants provided their written informed consent to participate in this study.

## Author contributions

SJW: Conceptualization, Data curation, Formal analysis, Funding acquisition, Investigation, Methodology, Project administration, Resources, Supervision, Validation, Visualization, Writing—original draft, Writing—review & editing. AC: Conceptualization, Funding acquisition, Validation, Writing—original draft. SLW: Conceptualization, Funding acquisition, Methodology, Validation, Writing—original draft. CC: Conceptualization, Formal analysis, Funding acquisition, Writing—original draft. MP: Conceptualization, Funding acquisition, Methodology, Validation, Writing—original draft.
