# Supplementary material for: Effectiveness and cost of integrated cognitive and balance training for balance and falls in cerebellar ataxia: a blinded two-arm parallel group RCT
Source: Front Neurol. 2024 Jan 19;14:1267099. doi: 10.3389/fneur.2023.1267099 (PMC10834731; doi:10.3389/fneur.2023.1267099)
Supplement: Supplementary file 1 [file Table_1.DOCX]

**Appendix 1**: Mean and standard deviation of the outcome measures at T1, T2, T3 and T4 assessment timepoints. Missing values replaced.

| Outcome measure (max score) | | | **T1 (Baseline 1)** | | **T2 (Baseline 2)** | | **T3 (Post intervention)** | | **T4 (Follow-up)** | |
| --- | --- | --- | --- | --- | --- | --- | --- | --- | --- | --- |
| ***Measures of balance and falls, mean / percentage (SD)*** | | | | | | | | | | |
|  | | | Group 1 (16) | Group 2 (16) | Group 1 (16) | Group 2 (16) | Group 1 (16) | Group 2 (16) | Group 1 (16) | Group 2 (16) |
| TUG | | Dual-task cost (Physical- %) |  |  | 55.80(11.83) | 50.01 (3.95) | 41.54 (8.82) | 49.90 (8.38) | 46.43 (9.37) | 53.43 (8.26) |
|  |  | Dual-task cost (Cognitive- %) |  |  | 137.55 (174.66) | 127.24 (39.38) | 95.18 (23.58) | 109.99 (29.74) | 93.63 (25.16) | 105.58 (18.01) |
|  |  | Dual-TUG (Sec) |  |  | 32.52 (15.01) | 34.45 (6.17) | 26.87 (9.97) | 33.27 (6.53) | 31.98 (11.31) | 39.67 (10.35) |
| BBS (56) | | |  |  | 33.88 (15.60) | 32.53 (6.38) | 34.75 (10.62) | 33.0 (5.38)) | 35.50 (13.11) | 31.69 (6.84) |
| SARAbal (18) | | | 7.06 (5.59) | 9.73 (3.53) | 7.21 (2.91) | 9.53 (3.54) | 6.50 (2.65) | 8.53 (2.82) | 9.12 (4.98) | 9.86 (3.73) |
| SOT | SOM (100) | |  |  | 103.44 (1.80) | 102.25 (3.61) | 93.50 (6.22) | 98.21 (10.63) | 97.56 (4.59) | 96.11 (3.60) |
|  | VIS (100) | |  |  | 77.12 (14.23) | 80.91 (9.96) | 70.58 (89.12) | 89.12 (8.53) | 64.18 (81.13) | 81.13 (4.13) |
|  | VEST (100) | |  |  | 65.93 (16.86) | 66.31 (12.04) | 83.88 (2.34) | 85.07 (10.48) | 78.68 (7.42) | 81.85 (14.40) |
|  | COMP (100) | |  |  | 81.31 (17.09) | 85.73 (13.06) | 98.20 (2.66) | 83.01 (16.02) | 86.31 (11.57) | 86.25 (14.67) |
| LOS | RT-F (ms) | |  |  | 1.76 (0.13) | 1.71 (0.52) | 1.14 (0.34) | 2.24 (0.50) | 1.71 (0.17) | 2.40 (0.46) |
|  | MXE-F (100) | |  |  | 70.38 (17.29) | 66.67 (4.81) | 88.43 (13.15) | 74.58 (11.75) | 91.85 (15.57) | 70.84 (16.93) |
|  | RT-R (ms) | |  |  | 1.19 (0.39) | 1.10 (0.29) | 0.91 (0.04) | 1.09 (0.25) | 1.11 (0.29) | 1.17 (0.62) |
|  | MXE-R (100) | |  |  | 97.05 (17.93) | 97.97 (6.11) | 89.71 (11.42) | 91.43 (45.16) | 81.93 (8.14) | 84.63 (15.36) |
|  | RT-B (ms) | |  |  | 1.61 (0.73) | 1.68 (0.52) | 1.47 (0.16) | 1.97 (0.51) | 1.83 (0.49) | 1.85 (0.49) |
|  | MXE-B (100) | |  |  | 86.81 (8.94) | 92.12 (8.89) | 74.39 (3.52) | 82.74 (39.16) | 80.63 (19.53) | 76.42 (14.54) |
|  | RT-L (ms) | |  |  | 1.19 (0.56) | 1.30 (0.42) | 1.07 (0.35) | 0.93 (0.21) | 1.18 (0.32) | 1.14 (0.55) |
|  | MXE-L (100) | |  |  | 88.60 (16.17) | 95.16 (12.11) | 98.81 (7.78) | 96.54 (37.69) | 99.09 (7.14) | 89.67 (16.14) |
| Percentage of fallers (at least 1 fall in the past month) | | |  |  | 31.25 | 37.5 | 25 | 37.5 | 31.5 | 31.25 |
| Number of falls/ person-1 month | | |  |  | 0.5 | 0.56 | 0.35 | 0.39 | 0.31 | 0.37 |
| Number of near falls/ person- 1 month | | |  |  | 2.43 | 2.87 | 2.62 | 2.43 | 2.68 | 2.81 |
|  | ***Measures of disease severity, quality of life and cognition, mean (SD)*** | | | | | | | | | |
| SARA (46) | | | 17.81 (6.37) | 20.68 (8.25) | 21.66 (7.78) | 25.31 (12.75) | 18.41 (3.29) | 21.81 (6.22) | 19.18 (6.58) | 22.06 (4.99) |
| EQ5D5L | EQ-VAS (100)  QALY (1) | |  |  | 66.88 (9.81)  0.75 (0.18) | 68.44 (14.91)  0.71 (0.28) | 71.0 (5.63)  0.77 (0.19) | 76.44 (14.60)  0.72 (0.15) | 70.93 (9.16)  0.69 (0.09) | 76.25 (5.92)  0.71 (0.19) |
| HK-MOCA (30) | | |  |  | 23.68 (4.02) | 22.72 (4.46) | 23.12 (3.96) | 23.72 (4.28) | 23.93 (4.28) | 23.13 (3.34) |

T1- assessment timeline 1, T2- assessment timeline 2, T3- assessment timeline 3, T4- assessment timeline 4RT- reaction time, MXE- Maximal excursion, Som- somatosensory, Vis-visual, Vest-vestibular, Comp- composite, BBS- Berg balance scale, SARA-bal- Balance sub-component of the scale for the assessment and rating of ataxia, R-right, F-front, L-left, B-back, RT- Reaction time, MXE- Maximal excursion, LOS- Limits of stability, EQ-VAS- visual analogue scale of the Euro Qol questionnaire, SARA- scale for the assessment and rating of ataxia.

**Appendix 2:** Within group mean difference and 95% confidence interval across T2 to T3 indicating the effect of intervention and T3 to T4 indicating retention of treatment effect during follow-up. Mean difference expressed as T2- T3 and T3-T4.

| Outcome measures | Time points comparison | Group 1 | | Group 2 | |
| --- | --- | --- | --- | --- | --- |
|  |  | MD (CI) | P value | MD (CI) | P value |
| DTUG | T2 vs T3 | -6.18 (-10.93 to -1.45) | 0.01* | -1.51 (-6.41 to 3.38) | 0.53 |
|  | T3 vs T4 | 5.64 (1.24 to 10.03) | 0.01 | 6.71 (2.17 to 11.24) | <0.01 |
| DTUGCost-Physical | T2 vs T3 | -14.26 (-20.99 to -7.53) | <0.01 | -0.11 (-6.63 to 6.64) | 0.97 |
|  | T3 vs T4 | 4.89 (-1.06 to 10.84) | 0.10 | 3.53 (-2.25 to 9.29 | 0.22 |
| DTUGCost- Cognitive | T2 vs T3 | -42.36 (-110.79 to 26.06) | 0.21 | -17.25 (-83.64 to 49.13) | 0.60 |
|  | T3 vs T4 | -1.55 (-21.17 to 18.07) | 0.87 | -4.40 (-23.44 to 14.65) | 0.64 |
| SARAbal | T1 vs T2 | 0.15 (-2.32 to 2.63) | 0.89 | -0.20 (-2.76 to 2.36) | 0.87 |
|  | T2 vs T3 | -0.71 (-2.64 to 1.20) | 0.45 | -1.0 (-2.82 to 2.61) | 0.31 |
|  | T3 vs T4 | 2.65 (-0.51 to 5.30) | 0.06 | 1.33 (-0.14 3 to 4.09) | 0.33 |
| BBS | T2 vs T3 | 0.87 (-4.56 to 6.31 | 0.74 | 0.46 (-5.14 to 6.08) | 0.23 |
|  | T3 vs T4 | 0.75 (-4.58 to 6.08) | 0.77 | -1.66 (-7.17 to 3.84) | 0.54 |
| SOT-SOM | T2 vs T3 | -9.64 (-14.40 to -4.88) | <0.01 | -4.25 (-9.17 to 0.66) | 0.08 |
|  | T3 vs T4 | 2.14 (-2.55 to 6.84) | 0.35 | -2.14 (-6.84 to 2.55) | 0.35 |
| SOT-VIS | T2 vs T3 | -6.53 (-14.97 to 1.89) | 0.12 | 8.21 (-0.49 to 16.92) | 0.06 |
|  | T3 vs T4 | -6.40 (-12.92 to 0.12) | 0.06 | -7.98 (-14.72 to -1.24) | 0.02* |
| SOT-VEST | T2 vs T3 | 17.94 (9.98 TO 25.91) | <0.01 | 19.18 (10.96 to 27.41) | <0.01 |
|  | T3 vs T4 | -5.19 (-12.62 to 2.24) | 0.16 | -3.54 (-11.21 to 4.13) | 0.35 |
| SOT-COMP | T2 vs T3 | 14.75 (3.70 to 25.79) | 0.01 | 1.29 (-10.11 to 12.71) | 0.86 |
|  | T3 vs T4 | -9.75 (-18.07 to -1.07) | 0.02 | -0.77 (-9.73 to 8.18) | 0.86 |
| LOS_F_RT | T2 vs T3 | -0.62 (-0.81 to -0.43) | <0.01 | 0.51 (0.34 to 0.73) | <0.01 |
|  | T3 vs T4 | 0.56 (0.33 to 0.79) | <0.01 | 0.15 (-0.83 to 0.39) | 0.19 |
| LOS_F_MXE | T2 vs T3 | 18.04 (10.21 to 25.86) | <0.01 | 7.91 (-0.16 to 15.99) | 0.06 |
|  | T3 vs T4 | 3.42 (-6.41 to 13.25) | 0.48 | -3.74 ((-13.90 to 6.41) | 0.45 |
| LOS_R_RT | T2 vs T3 | -0.27 (-0.47 to 0.6) | 0.13 | -0.01 (-0.21 to 0.20) | 0.94 |
|  | T3 vs T4 | 0.20 (-0.06 to 0.47) | 0.13 | 0.07 (-0.20 to 0.35) | 0.13 |
| LOS_R_MXE | T2 vs T3 | -7.34 (-27.18 to 12.51) | 0.45 | -6.54 (-27.03 to 13.95) | 0.51 |
|  | T3 vs T4 | -7.78 (-23.38 to 7.82) | 0.31 | -6.79 (-22.91 to 9.32) | 0.39 |
| LOS_B_RT | T2 vs T3 | -0.13 (-0.61 to 0.33) | 0.55 | 0.36 (-0.11 to 0.83) | 0.12 |
|  | T3 vs T4 | 0.34 (-0.03 to 0.73) | 0.07 | -0.11 (-051 to 0.26) | 0.53 |
| LOS_B_MXE | T2 vs T3 | -12.41 (-27.33 to 2.43) | 0.09 | -4.06 (-18.98 to 10.85) | 0.58 |
|  | T3 vs T4 | 6.23 (-10.05 to 22.52) | 0.44 | -6.53 (-22.82 to 9.76) | 0.41 |
| LOS_L_RT | T2 vs T3 | -0.11 (-0.44 to 0.21) | 0.47 | -0.36 (-0.71 to -0.26) | 0.03 |
|  | T3 vs T4 | 0.11 (-0.15 to 0.36) | 0.41 | 0.21 (-0.05 to 0.47) | 0.10 |
| LOS_L_MXE | T2 vs T3 | 10.21 (-5.51 to 15.81) | 0.19 | 1.37 (-14.84 to 17.60) | 0.86 |
|  | T3 vs T4 | 0.28 (-15.81 to 16.37) | 0.97 | -6.86 (-23.48 to 9.75) | 0.41 |
| ***Measures of disease severity, quality of life and cognition, mean (SD)*** | | | | | |
| SARA | T1 vs T2 | 3.85 (-7.90 to 0.18 | 0.61 | -4.62 (-8.67 to -0.58) | 0.02 |
|  | T2 vs T3 | -3.26 (-8.40 to 1.83) | 0.20 | -3.50 (-8.61 to 1.65) | 0.17 |
|  | T2 vs T4 | -2.48 (-7.40 to 2.44) | 0.31 | -3.25 (-8.17 to 1.67) | 0.18 |
| MOCA | T2 vs T3 | -0.56 (-3.46 to 2.33) | 0.69 | 1 (-1.99 to 3.99) | 0.50 |
|  | T3 vs T4 | 0.81 (-1.72 o 3.35) | 0.51 | -0.60 (-3.22 to 2.02) | 0.64 |
| EQ_VAS | T2 vs T3 | 4.12 (-3.95 to 12.20) | 0.30 | 8.0 (-0.80 to 16.08) | 0.05* |
|  | T3 vs T4 | -0.62 (-6.91 to 6.78) | 0.98 | -0.18 (-7.03 to 6.65) | 0.95 |
| QALY | T2 vs T3 | 0.01 (-0.10 to 0.29) | 0.79 | 0.14 (-0.10 to 0.12) | 0.79 |
|  | T3 vs T4 | -0.69 (-0.18 to 0.04) | 0.23 | -0.01 (-0.12 to 0.10) | 0.87 |

T1 assessment timeline 1, T2- assessment timeline 2, T3- assessment timeline 3, T4- assessment timeline 4, RT- reaction time, MXE- Maximal excursion, Som- somatosensory, Vis-visual, Vest-vestibular, Comp- composite, BBS- Berg balance scale, SARA-bal- Balance sub-component of the scale for the assessment and rating of ataxia, R-right, F-front, L-left, B-back, RT- Reaction time, MXE- Maximal excursion, LOS- Limits of stability, EQ-VAS- visual analogue scale of the Euro Qol questionnaire, SARA- scale for the assessment and rating of ataxia.
